# Anesthetic Management in Peripartum Cardiomyopathy: A Contemporary Review

**DOI:** 10.7759/cureus.33159

**Published:** 2022-12-31

**Authors:** Aishwarya Nayak, Sanjot Ninave, Surekha Tayade, Harshal Tayade

**Affiliations:** 1 Anaesthesiology, Jawaharlal Nehru Medical College, Datta Meghe Institute of Higher Education and Research (Deemed to be University), Wardha, IND; 2 Obstetrics and Gynecology, Jawaharlal Nehru Medical College, Datta Meghe Institute of Higher Education and Research (Deemed to be University), Wardha, IND; 3 General Surgery, Mahatma Gandhi Institute of Medical Sciences, Wardha, IND

**Keywords:** high-risk pregnancy, anesthesia, peripartum cardiomyopathy, obstetric anesthesia in critical illness, anesthesia in ppcm, anesthesia in high risk pregnancy, obstetric anesthesia

## Abstract

Peripartum cardiomyopathy (PPCM) is an uncommon disorder of the cardiovascular system and is linked to high rates of morbidity and mortality. It is an idiopathic condition characterized by left ventricular systolic dysfunction with an ejection fraction of approximately 45% near the end of pregnancy or immediately after delivery. Anesthesia management in these women is challenging due to low physiological reserve and potential negative effects on the fetus. To ensure that mother and child are supported safely through delivery, careful anesthesia control is required. Here, in this review article, we discuss the anesthetic implications in preoperative, operative, and postoperative phases in women with perioperative cardiomyopathy undergoing vaginal delivery or cesarean section.

## Introduction and background

Peripartum cardiomyopathy (PPCM) is an uncommon, potentially fatal clinical condition with no recognized cause. The definition has four requirements: (a) the onset of heart failure within five months of delivery or in the final month of pregnancy; (b) the heart failure has no known etiology; (c) there is no obvious cardiac illness before the last month of pregnancy; and (d) there should be echocardiographic evidence of left ventricular systolic dysfunction (ejection fraction of 45% and reduced fractional shortening) [1,2]. Thus, it is a diagnosis of exclusion. Proper anesthetic management is imperative to assure the safety of the mother and baby through childbirth. Neuraxial anesthetic use must be controlled to maintain hemodynamic stability, depending upon the capacity of the pregnant woman to handle the physiological stress of pregnancy. In this review, we shall address epidemiology, risk factors, and anesthetic management in the perioperative and postoperative period for women with PPCM, undergoing vaginal delivery or cesarean section.

## Review

Methods

**Figure 1:**
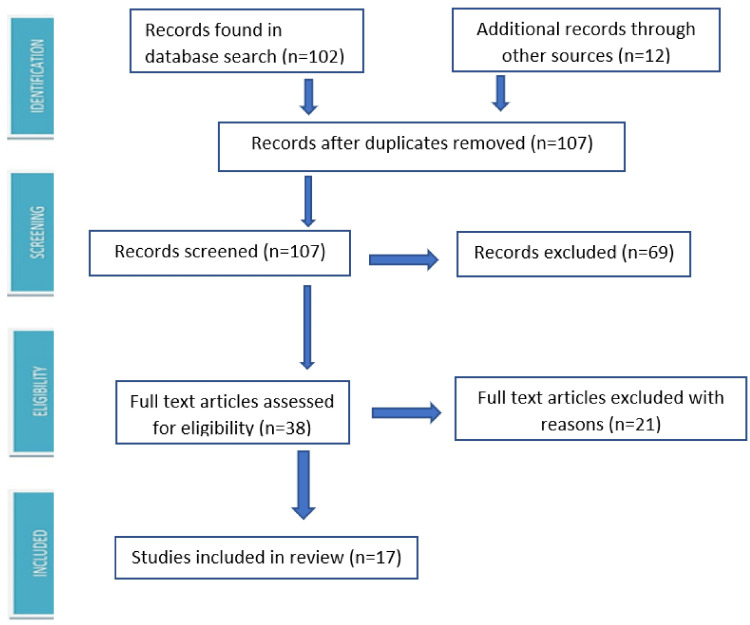
PRISMA flow diagram PRISMA: Preferred Reporting Items for Systematic Reviews and Meta-Analyses.

We searched PubMed, MEDLINE, Embase, ISI Web of Science, and Google Scholar databases using the Medical Subject Heading (MeSH) terms, including anesthesia, pregnancy, and peripartum cardiomyopathy, and found 102 articles from 1985 to 2022. When we limited our search to the last 12 years, i.e., from 2011 to 2022, we were able to obtain 17 pertinent publications (Figure 1). The articles reporting the anesthetic management in the perioperative and postoperative period in women with PPCM were included. Records that did not address anesthesia considerations in PPCM and those without the availability of full text were excluded. The pertinent articles included in the review are shown in Table 1.

**Table 1:** Relevant articles included in the review PPCM: peripartum cardiomyopathy.

S. No.	Author	Year	Place	Findings
1	Osinaike et al. [3]	2011	South Africa	A 30-year-old woman with PPCM, requiring an emergency C-section, was successfully managed with combined spinal-epidural anesthesia, using low-dose fentanyl for spinal anesthesia.
2	Zeng et al. [4]	2012	China	For the cesarean section, lidocaine was used with a continuous epidural anesthetic while the patient was sitting.
3	Löser et al. [5]	2013	Hamburg, Germany	A heavily pregnant patient with the signs of acute severe heart failure delivered under peri-dural anesthesia under heart-pulmonary machine readiness.
4	Basak et al. [6]	2013	Canada	A woman with severe pre-eclampsia who had acute onset heart failure secondary to PPCM underwent cesarean delivery under general anesthesia with rapid sequence intubation.
5	Dutt et al. [7]	2013	India	In these situations, it is wise to use titrated epidural anesthetic with careful fluid and inotropic support.
6	Sumikura et al. [8]	2014	Tokyo	Regional anesthetic or general anesthesia can be used during cesarean sections; however, low-dose combined spinal-epidural analgesia has been shown to be a reliable option.
7	Ashikhmina et al. [9]	2015	USA	Women who do not have orthopedic, neurological, or coagulopathy-related restrictions can receive neuraxial labor analgesia. After PCEA (patient-controlled epidural analgesia) dosage or infusion, no immediate hemodynamic impairment was seen. In surgical patients with significant left ventricular outflow tract obstruction, the use of an epidural catheter allows intrathecal block and administration of local anesthesia in small doses. This avoids hemodynamic instability associated with spinal anesthesia.
8	Munro et al. [10]	2015	Canada	Preload, afterload, and the variations in cardiac output caused by labor can all be reduced with regional anesthesia.
9	Ituk et al. [11]	2015	USA	Out of 25 patients, nine had vaginal deliveries with epidural analgesia started either before labor was induced or during the latent stage of labor. Three women received general anesthesia, five women underwent epidural anesthesia, and seven women underwent combined spinal-epidural anesthesia for their cesarean deliveries. During the induction of general anesthesia, one patient experienced a cardiac arrest and was successfully revived. There were no neonatal or maternal fatalities.
10	Bauersachs et al. [12]	2016	Germany	Regardless of gestational age, patients who experience hemodynamic instability despite treatment should have an urgent delivery. The use of a skilled interdisciplinary team and a cesarean section with combined spinal and epidural analgesia are advised.
11	Beaudry et al. [13]	2016	New York, USA	A 24-year-old woman underwent cesarean delivery under epidural anesthesia with the use of intraoperative non-invasive cardiac output monitoring. She had an aortic arch, aortic valve, mitral valve replacements, and a left ventricular ejection fraction of 37%.
12	Hoy et al. [14]	2017	England, UK	A combined spinal epidural was used to induce a 33-year-old primigravida who had severe sepsis, severe pre-eclampsia, peripartum cardiomyopathy, hemolysis, elevated liver enzymes, and low platelets.
13	Mikami et al. [15]	2018	Japan	A cesarean section was performed on a 28-year-old woman who was gravida 3 and para 2 at 35 weeks and 6-7 days of gestation and had acute severe peripartum cardiomyopathy (ejection fraction: 10%) under general anesthesia and mechanical circulatory support.
14	Sivakumar et al. [16]	2019	India	A bolus of 10 ml of 0.0625% bupivacaine with 2 mcg per ml fentanyl was given to provide epidural labor analgesia.
15	Baldini et al. [17]	2019	Italy	The use of combined spinal epidural (CSE) procedures is crucial for treating PPMC patients; single-shot spinal anesthesia is not recommended to prevent the administration of large medication doses for neuraxial anesthesia during CS. If required, we can inject some painkillers and provide low drug doses (LDLA) utilizing the CSE procedure (e.g. paracetamol).
16	Sahu et al. [18]	2021	India	A 28-year-old woman parturient presenting at 37 weeks of gestation for cesarean section with recently diagnosed peripartum cardiomyopathy (ejection fraction of 28%) complicated by severe preeclampsia developed cardiac failure just before the induction of anesthesia was resuscitated and operated under general anesthesia.
17	Ng et al. [19]	2022	Taiwan	General anesthesia was used to induce PPCM in a pregnant woman having a cesarean delivery who had influenza A and pneumonia.

Risk factors and etiopathology

PPCM is a heterogenous condition and the exact etiology is not known. This is most likely due to its multifactorial nature and also because its definition focuses on a particular time frame during pregnancy, to achieve a diagnosis, instead of relating to a certain pathophysiological process.

Multiparity, advanced maternal age (>30 years old), multiple pregnancies, preeclampsia, gestational hypertension, and women of African ethnicity are potential risk factors [20-23]. Wang et al. suggested that multiparity (> three children), twin pregnancies, preeclampsia, and infrequently other hypertensive illnesses are significant correlations [24]. Among women outside of European origin, a substantial correlation between PPCM and HIV infection appears to exist. PPCM has been reported in 24-37% of young primigravidae [25-27]. Long-term tocolysis and African race have also been implicated as determinants [26,28-30]. Selenium and zinc deficiency and substance abuse are other risk factors [22].

Infectious, inflammatory, hormonal, autoimmune, pro-apoptotic, hemodynamic, and genetic causes have all been proposed. In fact, several therapies based on these hypotheses, such as intravenous immunoglobulin, have been attempted but have not been particularly successful [31]. Recently, a hereditary propensity to PPCM has been reported (Table 2) [32-34].

**Table 2:** Risk factors for peripartum cardiomyopathy

Maternal factors	Fetal factors	Antepartum and intrapartum	Other causes
Increased age (>30 years)	Multiple pregnancies	Use of prolonged tocolysis	Infectious
High parity	Artificial reproductive techniques	High prolactin levels	Inflammatory
African ethnicity			Hormonal
Preeclampsia			Autoimmune
Selenium and zinc deficiency			Pro-apoptotic
Zaria region of Nigeria			Hemodynamic
HIV infection			Genetic
Previous peripartum cardiomyopathy			
Previous hypertensive disorder			

If you look at the pathophysiology, oxidative stress along with decreased angiogenesis appears to be the underlying mechanism [35]. Hilfiker et al. reported that high blood pressure, atypical reactions to hemodynamic alteration, viral affliction, and deficient nutrients may all contribute to this oxidative stress [36]. Maladaptive response to hemodynamic stresses of pregnancy may lead to PPCM [20]. There is a supposition that an immunologically mediated mechanism involving maternal antibody sensitization to fetal cells could be a possibility. These could sensitize maternal antibodies to myocardial epitopes, explaining why some PPCM patients have antibodies to cardiac myosin heavy chains [29]. Hilfiker et al. also proposed that prolactin metabolism may also play a role in the disruption of cardiomyocyte angiogenesis that leads to cardiac failure [36]. Lampert et al. found a link between prolonged tocolysis and PPCM [37].

Obstetric considerations and type of anesthesia in PPCM

A multidisciplinary approach has to be planned for appropriate obstetric and anesthesia management. The team should include all stakeholders since the diagnosis of PPCM can be antepartum, intrapartum, or postpartum. Along with the obstetrician, other teammates include most importantly the anesthesiologist, cardiologist, and critical care specialist. An expert neonatologist and a competent team of nurses are necessary as add-ons.

As PPCM is basically a diagnosis of exclusion, we should consider a range of disorders in differential diagnosis, especially if the event is acute [20]. It is imperative to rule out myocardial infarction (MI), dissecting aorta, viral myocarditis, and stenosis of the renal artery. Investigations include serum troponin and brain natriuretic peptide, kidney functions, and electro and echocardiography [33]. The infiltrative disease of the heart can be diagnosed by an MRI. Furthermore, angiography may be needed to pick up MI. A cardiac biopsy is, however, optional. In women with PPCM, HIV tests may have to be repeated, despite being negative in prenatal booking [20].

First and foremost, the team should decide whether there is a stable heart or a decompensated heart. In case of stable heart failure, the team may decide on the use of conventional heart failure medications and continuation of the pregnancy till term. Fetal surveillance has to be frequent to detect complications early and guide interventions. In stable heart failure without obstetric indications, vaginal delivery is preferred [33]. To avoid sympathetic stress and vasoconstriction, epidural labor analgesia should be used. Furthermore, a generous episiotomy, ventouse application, or outlet forceps can be used to shorten the second stage of labor. To avoid neurological complications, anticoagulation must be discontinued depending on the pharmacokinetics during epidural catheter insertion [29]. Vaginal delivery with assistance and cesarean section is only used for obstetric reasons. Oxygen supplementation may or may not be needed in stable cases.

Hypoxia, cardiogenic shock, and hypotension are deleterious to the fetus, as they reduce maternal oxygen delivery. Emergent delivery planned with the help of an attending neonatologist will improve fetal outcomes [22].

In patients undergoing emergency cesarean delivery or receiving anticoagulant therapy, general anesthesia appears to be appropriate. Epidural local anesthetic needs to be delivered in small incremental doses. Alternatively, spinal anesthesia may be instituted by adding fentanyl to a local anesthetic. Additionally, neuraxial anesthesia has a major benefit over general anesthesia in terms of long-term pain relief. The choice between regional and general anesthesia would depend on prenatal anticoagulant use and the need for emergent delivery [22].

**Figure 2:**
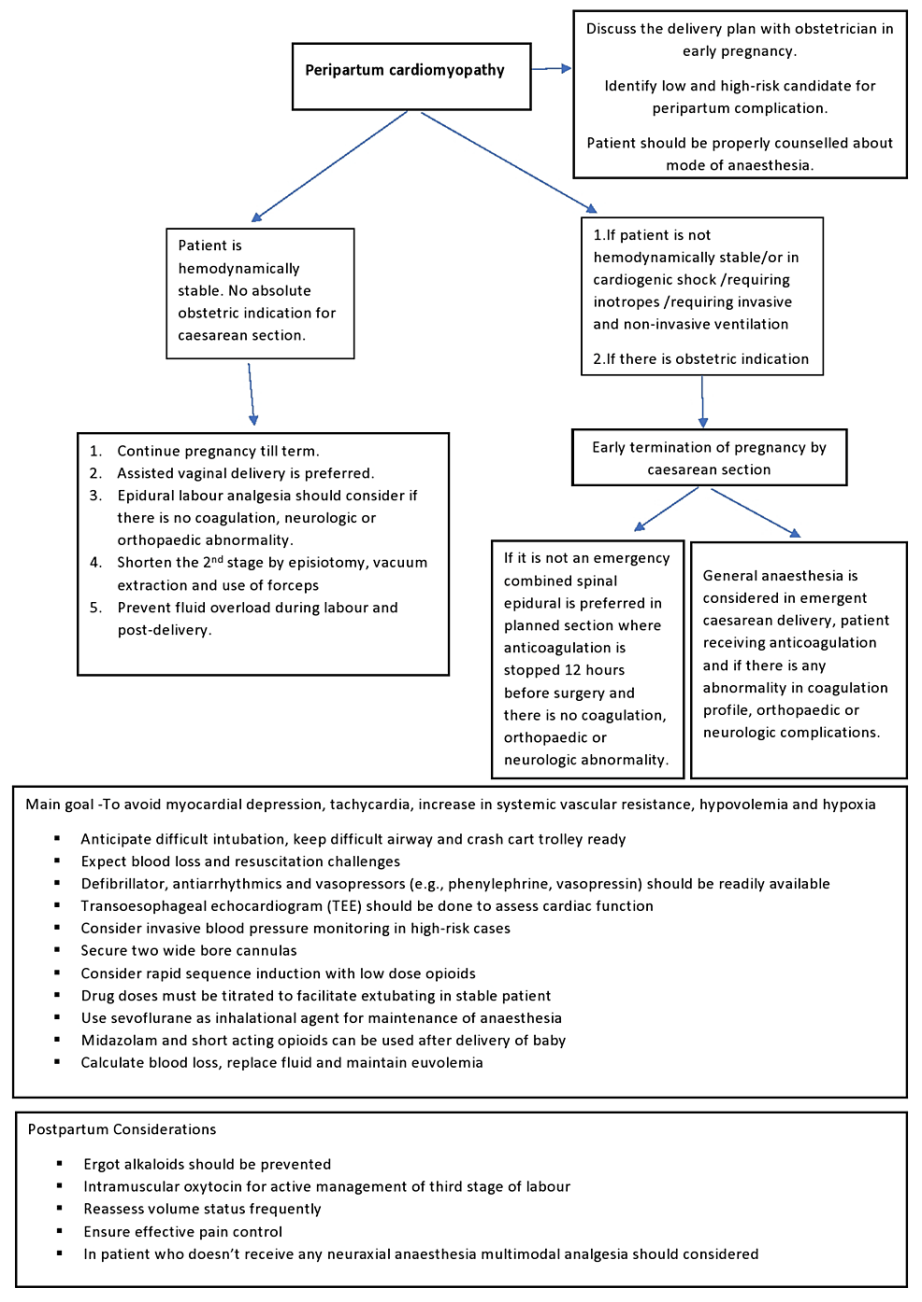
Managing peripartum cardiomyopathy with anesthetic considerations

Inotropes are required for cardiogenic shock. Ventilation is required, either invasive or noninvasive. Mechanical assist devices are required. Pregnancy termination by cesarean section should be considered. General anesthesia based on opioids should be administered. Neonatal evaluation and neonatal intensive care unit (NICU) treatment should be considered. The following flow chart outlines the management (Figure 2).

Anesthetic considerations in peripartum cardiomyopathy

Given their diminished physiological reserves and potential negative consequences on the unborn child, the anesthetic management of these patients can be difficult. Any patient who has PPCM should almost probably have an anesthetist review them and may need emergency measures [20].

The same operative principles should be followed in the case of proposed vaginal birth (Table 3).

**Table 3:** Preoperative and intraoperative anesthetic considerations

Preoperative considerations	Intra-operative considerations
1. An obstetrician should be consulted early on, to plan the delivery.	1. Identify potential peripartum complications in high- and low-risk individuals such as fetal factors, preeclampsia, high left ventricular outflow tract gradients, regurgitant mitral valve, the systolic anterior motion of the mitral valve, cardiac arrhythmias, and reduced heart muscle contractibility.
2. Plan ahead for surgical anesthesia as well as neuraxial labor analgesia.	2. For high-risk individuals, think about invasive arterial blood pressure monitoring.
3. Identify any contraindications to neuraxial anesthesia.	3. To determine preload, take into account transesophageal echocardiogram or non-invasive cardiac output monitoring.
4. The woman should be given alternative analgesic options with the possibility of general anesthesia if there are any contraindications.	4. Avoid both overhydration and hypovolemia by strict watch on fluid balance and replacement of lost fluid.
5. Obtain an opinion from a cardiologist and have the necessary tests performed, such as a transesophageal echocardiogram (TTE) to check for left ventricular outflow tract gradients both at rest and when stimulated, ventricular and valvular dysfunction, and Holter electrocardiogram with stress exam if needed.	5. A carefully titrated epidural catheter is recommended for labor analgesia to ensure comfort.
	6. Instead of using a single-shot spinal anesthetic for scheduled cesarean delivery, use a sequential combined spinal epidural (CSE) approach.
	7. Antiarrhythmics and a defibrillator should be available when required.
	8. Assure ready availability of phenylephrine and vasopressin.
	9. Beta-1 agonists and sympathomimetics need to be avoided to decrease left ventricular outflow tract obstruction.

Hemodynamic considerations in neuraxial anesthesia in PPCM

Managing hemodynamics around the time of birth in women having PPCM is challenging, as the gravid uterus compresses the vena cava, and the need for adequate blood pressure to maintain a placental blood supply and the possibility of excessive blood loss lead to depleted intravascular space. Both epidural and spinal anesthesia cause acute vasodilation, with reduced preload and afterload, leading to a fall in blood pressure. This worsens in PPCM as cardiac contractility is poor and cannot compensate for the hypotension. Epidural analgesia, with the incremental anesthetic bolus, administered in the early first and second stages of labor is advantageous in PPCM, as it provides adequate neuraxial block over an extended time period. This also takes care of maternal fatigue by reducing pain.

It is best to place an arterial line before topping up an epidural or giving spinal anesthesia unless a cesarean section is emergent. In case of severe decompensated heart failure, general anesthesia is considered safer, and neuraxial needs to be completely avoided. An experienced and senior anesthetist should preferably manage the case [22].

We need to particularly avoid acidosis, hypoxemia, and anemia, as all of these can lead to dysfunction of the heart muscle. Blood loss should be closely monitored by regularly weighing the sponges and maintaining an input-output chart. Furthermore, the use of excessive crystalloids for resuscitation may contribute to third space loss and pulmonary edema, and thus should be restricted. Arrhythmias may be treated with appropriate medications and electrotherapy. Digoxin and amiodarone are better options as compared to beta blockers. Calcium channel blockers may cause tocolysis and relaxation of uterine musculature, leading to atonic hemorrhage, and are best avoided.

## Conclusions

PPCM is a rare but life-threatening condition that must be managed by experienced clinicians. The goal of anesthesia is to maintain normal physiology in terms of acid-base balance, hypoxia, and anemia. To ensure adequate preload for maintaining cardiac output, intravascular volume needs to be managed with utmost care. Excessive crystalloid fluid replacement, however, can cause pulmonary edema and decompensated cardiac failure, and its use should be avoided. Perioperative fluid management has to be continuous and meticulous for successful outcomes in women with PPCM.

There is no established anesthetic treatment protocol, so the decision between the neuraxial block and general anesthesia must be individualized. A central line for the administration of vasopressors and inotropes, along with an epidural with incremental top-ups, invasive blood pressure monitoring, and possibly other measures, may be a better choice. These may be dictated by the physiological cardiac reserve of the patient and the need for urgent delivery of the baby. Advanced hemodynamic monitors such as cardiac output monitoring devices are particularly helpful in decisions regarding appropriate volume replacement, and the use of vasopressors and inotropes when needed. An experienced team and meticulous planning contribute to successful outcomes in PPCM. Planning early for labor and delivery strategies is imperative.
